# Mesenchymal stem cells as a novel vaccine platform

**DOI:** 10.3389/fcimb.2012.00140

**Published:** 2012-11-16

**Authors:** Suzanne L. Tomchuck, Elizabeth B. Norton, Robert F. Garry, Bruce A. Bunnell, Cindy A. Morris, Lucy C. Freytag, John D. Clements

**Affiliations:** ^1^Department of Microbiology and Immunology, Tulane University School of MedicineNew Orleans, LA, USA; ^2^Tulane Center for Stem Cell Research and Regenerative Medicine, Tulane University School of MedicineNew Orleans, LA, USA; ^3^Department of Pharmacology, Tulane University School of MedicineNew Orleans, LA, USA

**Keywords:** MSC, vaccination, adaptive immunity, antibodies, antigen delivery

## Abstract

Vaccines are the most efficient and cost-effective means of preventing infectious disease. However, traditional vaccine approaches have thus far failed to provide protection against human immunodeficiency virus (HIV), tuberculosis, malaria, and many other diseases. New approaches to vaccine development are needed to address some of these intractable problems. In this report, we review the literature identifying stimulatory effects of mesenchymal stem cells (MSC) on immune responses and explore the potential for MSC as a novel, universal vaccination platform. MSC are unique bone marrow-derived multipotent progenitor cells that are presently being exploited as gene therapy vectors for a variety of conditions, including cancer and autoimmune diseases. Although MSC are predominantly known for anti-inflammatory properties during allogeneic MSC transplant, there is evidence that MSC can actually promote adaptive immunity under certain settings. MSC have also demonstrated some success in anti-cancer therapeutic vaccines and anti-microbial prophylactic vaccines, as we report, for the first time, the ability of modified MSC to express and secrete a viral antigen that stimulates antigen-specific antibody production *in vivo.* We hypothesize that the unique properties of modified MSC may enable MSC to serve as an unconventional but innovative, vaccine platform. Such a platform would be capable of expressing hundreds of proteins, thereby generating a broad array of epitopes with correct post-translational processing, mimicking natural infection. By stimulating immunity to a combination of epitopes, it may be possible to develop prophylactic and even therapeutic vaccines to tackle major health problems including those of non-microbial and microbial origin, including cancer, or an infectious disease like HIV, where traditional vaccination approaches have failed.

## Introduction

**Figure 1:**
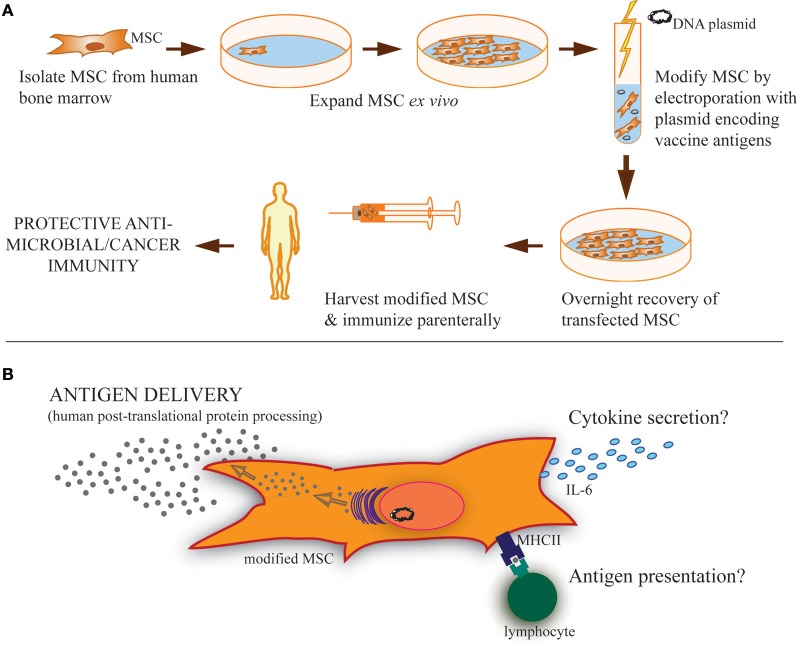
**Strategy of modified MSC vaccination and possible MSC functions during vaccination. (A)** MSC isolated from the bone marrow of human donors can be expanded in culture and modified by transfection using antigen(s)-encoding plasmid to express and secrete soluble proteins, including both cancer and microbial antigens. Parenteral immunization of these modified MSC could then provide protective immunity. **(B)** These modified MSC may carry out several possible functions after vaccination. Primarily, it is expected that they serve as antigen delivery vehicles or even antigen depots following immunization. Based on the literature, it is clear that MSC can also take a more active role in induction of adaptive immunity, including cytokine secretion, like IL-6, and/or antigen presentation through phagocytosis and MHC-loading of antigen for presentation to lymphocytes expressing cognate T-cell/B-cell receptors. These immunostimulatory functions may also be involved in MSC-based vaccinations.

While vaccination programs have clear documented success in controlling many diseases, there has been a failure to generate effective, long-term immunity against certain major pathogens. On the other hand, in carcinogenic situations there is an urgent need to develop therapies that promote the host immune system to target and destroy cancerous tumors and metastases. Mesenchymal stem cells (MSC) are unique bone marrow-derived multipotent progenitor cells that are presently being exploited as gene therapy vectors for a variety of conditions, including cancer and autoimmune diseases (Klopp et al., 2007; Le Blanc and Ringden, 2007; Spaeth et al., 2008; Bergfeld and Declerck, 2010; Chen et al., 2010; Liang et al., 2010; Martino et al., 2010; Panes et al., 2010). Although MSC are predominantly known for anti-inflammatory properties during allogeneic MSC transplant, there is evidence that MSC can actually promote adaptive immunity under certain settings. We hypothesize that the unique properties of modified MSC may enable these cells to serve as an unconventional but innovative, vaccine platform (described in Figure 1A). Such a platform would be capable of expressing hundreds of proteins, thereby generating a broad array of epitopes with correct post-translational processing, mimicking natural infection. In this report, we review the literature supporting our hypothesis by identifying stimulatory effects of MSC on immune responses and demonstrate, as proof of concept, the ability of modified MSC to express and secrete a viral antigen that stimulates antigen-specific antibody production *in vivo*.

## Current problems in vaccination strategies

### The ideal vaccine

Vaccines are the most efficient and cost-effective means of preventing infectious disease. Vaccines have already demonstrated transformative potential in eradicating one devastating disease, smallpox, while offering the ability to control other diseases, including diphtheria, polio, and measles, that formerly caused widespread morbidity and mortality. The development of vaccines involves the testing of an attenuated or inactivated version of the pathogen or identification of a pathogen component(s) (i.e., subunit, toxoid, and virus-like particle) that elicits an immune response that protects recipients from disease when they are exposed to the actual pathogen. In an ideal world a single vaccine would be able to target all major human pathogens (versatile), elicit strong protective immunity to these pathogens (robust) without inducing unwanted side-effects (safe), and still be fairly inexpensive to produce per dose (cost-effective). In the case of viruses or host-cell produced proteins, vaccine production that includes human post-translational processing, mimicking natural infection, will likely prove to be superior to bacterial or other expression systems.

### Failures of traditional strategies

Traditional vaccine approaches have thus far failed to provide protection against human immunodeficiency virus (HIV), tuberculosis, malaria, and many other diseases, including dengue, herpes, and even the common cold. The reasons why traditional vaccine approaches have not been successful for these diseases are complex and varied. For example, HIV integrates functional proviral genomes into the DNA of host cells, thereby establishing latency or persistence. Once latency/persistence is established, it has not been possible to eradicate HIV, even with highly active antiretroviral therapy. Clearly, new approaches to vaccine development are needed to address HIV and other intractable vaccine challenges.

### Non-traditional vaccine strategies

Newer alternative immunization approaches include both DNA and cellular vaccines. DNA vaccines involve the transfection of cells at the tissue site of vaccination with an antigen encoding plasmid that allows local cells (i.e., myocytes) to produce the vaccine antigen *in situ*. Cellular vaccines use the direct transfer of pre-pulsed or transfected host cells [i.e., dendritic cells (DC)] expressing or presenting the vaccine antigen. The advantage of these approaches is that vaccine antigens are produced *in vivo* and are readily available for immunological processing. Despite numerous reports of successful pre-clinical testing, both such approaches have hit stumbling blocks. DNA vaccination studies in humans show poor efficacy, which was linked to innate differences between mice and humans (Cavenaugh et al., 2011; Wang et al., 2011). DC vaccination strategies have shown limited clinical success for therapeutic cancer vaccinations and have high production costs due to necessary individual tailoring (Bhargava et al., 2012; Palucka and Banchereau, 2012).

## MSC-based cellular therapeutics

MSC are unique bone marrow-derived multipotent stem cells that are presently being exploited as gene therapy vectors for a variety of conditions, including cancer and autoimmune diseases (Klopp et al., 2007; Le Blanc and Ringden, 2007; Spaeth et al., 2008; Bergfeld and Declerck, 2010; Liang et al., 2010; Lim et al., 2010; Martino et al., 2010; Panes et al., 2010). These progenitor cells are known to migrate to sites of inflammation, infection, tissue injury, and tumors where they immunomodulate the microenvironment through cell-to-cell contact and the release of soluble factors, thus facilitating the repair of damaged tissue (Aggarwal and Pittenger, 2005; Gotherstrom, 2007). For more information see recent reviews on the immunomodulatory properties of MSC therapy (Le Blanc and Ringden, 2007; Stagg, 2007; Tolar et al., 2007; Franquesa et al., 2012; Yi and Song, 2012).

A main contributing factor to therapeutics designed around MSC is the ease of MSC isolation and expansion in culture. Theoretically, a single bone marrow harvest of MSC may yield sufficient MSC for thousands of clinical applications, due to their inherent expansion capability (Newman et al., 2009). Such expansion potential greatly enhances the GMP manufacturing capability of using MSC for clinical applications and has lower production costs when compared to other cell types.

MSC have been successfully transplanted into allogeneic hosts in a variety of clinical and pre-clinical settings (Di Nicola et al., 2002; Meisel et al., 2004; Aggarwal and Pittenger, 2005; Chen et al., 2006; Corcione et al., 2006; Sotiropoulou et al., 2006; Uccelli et al., 2007; Raffaghello et al., 2008). These donor MSC often promote immunotolerance (Potian et al., 2003; Aggarwal and Pittenger, 2005), including the inhibition of graft-versus-host disease (GvHD) that can develop after cell or tissue transplantation from a major histocompatibility complex (MHC)-mismatched donor (Ringden et al., 2006; Wernicke et al., 2011). The diminished GvHD symptoms after MSC transfer has been due to direct MSC inhibition of T and B cell proliferation, resting natural killer cell cytotoxicity, and DC maturation (reviewed in Uccelli et al., 2008). Although, in contrast, at least one study has reported generation of antibodies against transplanted allogeneic MSC (Sundin et al., 2007). Nevertheless, the ability to prevent GvHD also suggests that MSC expressing foreign antigen might have an advantage over other cell types (i.e., DC) during a cellular vaccination in selectively inducing immune responses to only the foreign antigen(s) expressed by MSC and not specifically the donor MSC. Thereby, MSC as the cellular base for an alternative vaccination strategy may save on production time and costs associated with necessary HLA matching if other cell types were used.

In order to enhance their immunomodulatory properties, the use of modified MSC is also being explored *in vivo* (Choi et al., 2008; Sasaki et al., 2009; Kumar et al., 2010; Klinge et al., 2011). MSC can be easily transfected with protein encoding plasmids, for transient protein expression or a more long-term, stable transfection and prolonged protein expression. MSC, transduced to overproduce IL-10, suppressed collagen-induced arthritis in a mouse model (Choi et al., 2008). In addition, MSC expressing glucagon-like peptide-1 transplanted into an Alzheimer's disease mouse model led to a decrease in A-beta deposition in the brain (Klinge et al., 2011). In an osteopenia mouse model, mice receiving transduced MSC that had stable expression of bone morphogenetic protein had increased bone density (Kumar et al., 2010). In a rat model for spinal cord injury, rats treated with MSC stably overexpressing of brain-derived neurotrophic factor had a better overall outcome than rats administered MSC alone (Sasaki et al., 2009). Lastly, in a rat model for bladder outlet obstruction, rats receiving transduced MSC with stable overexpression of hepatocyte growth factor had decreased collagen accumulation in the bladder (Song et al., 2012). These studies indicate that modified MSC are a useful and feasible vehicle for protein expression/delivery to target various diseases and tissues.

## MSC as a vaccine platform

An MSC delivery platform is similar to that of a DNA vaccine or cellular vaccine in that the antigen is expressed through DNA transfection and delivered by an *ex vivo* cultured cell. It may be that this MSC strategy improves on problems that have occurred with DNA and DC-based vaccinations. There are two basic uses of such an MSC platform, as an anti-cancer therapeutic vaccine or as an anti-microbial prophylactic vaccine, discussed in more detail below.

### Anti-cancer therapeutic vaccines

MSC have been studied as a delivery vehicle for anti-cancer therapeutics due to their innate tendency to home to tumor microenvironments, and is thoroughly reviewed in Loebinger and Janes (2010). MSC have also been used to promote apoptosis of tumorigenic cells through the expression of IFNα or IFNγ (Li et al., 2006; Ren et al., 2008). Additionally, MSC have recently been explored as novel, vaccine platform in the prevention and inhibition of tumorigenesis and metastasis. A unique study by Wei et al. examined the use of human papilloma virus (HPV)-immortalized MSC that express the HPV proteins E6/E7 combined with a modified E7 fusion protein vaccine in a mouse tumor model where metastatic fibrosarcoma cells were administered (Wei et al., 2011). This group found that only when mice were immunized with both the E7-expressing MSC and modified E7 protein vaccine did mice show a decrease in tumor growth, and an E7-specific antibody response. Mice receiving either MSC or protein vaccine alone were not able to raise an anti-E7 response or inhibit tumor growth of metastatic sarcoma. The limitation of this interesting approach is that it can only be used as an anti-cancer therapeutic and not as a universal cancer preventative, as individual tumors have unique antigen expression. In addition, a long-term safety examination of these immortalized MSC/protein vaccine therapy in cancer-free mice is warranted. Although these immortalized MSC were previously determined to be non-tumorigenic (Hung et al., 2004), they persisted in mice longer than 21 days, unlike primary MSC (i.e., non-immortalized) which are only detectable for a very short time after administration (Gao et al., 2001; Abraham et al., 2004; Ohtaki et al., 2008; Prockop, 2009). Thus, there may be unforeseen outcomes in the long term (i.e., outcompeting with endogenous MSC and differing immunomodulatory abilities, which were not assessed in this study) with the use of immortalized MSC even if they prove to be non-malignant. Other studies have indicated that immortalized MSC can become tumorigenic, and thus must be carefully studied to determine if they are indeed safe for use (Rubio et al., 2005; Phinney and Prockop, 2007; Tolar et al., 2007).

### Anti-microbial prophylactic vaccines

**Figure 2:**
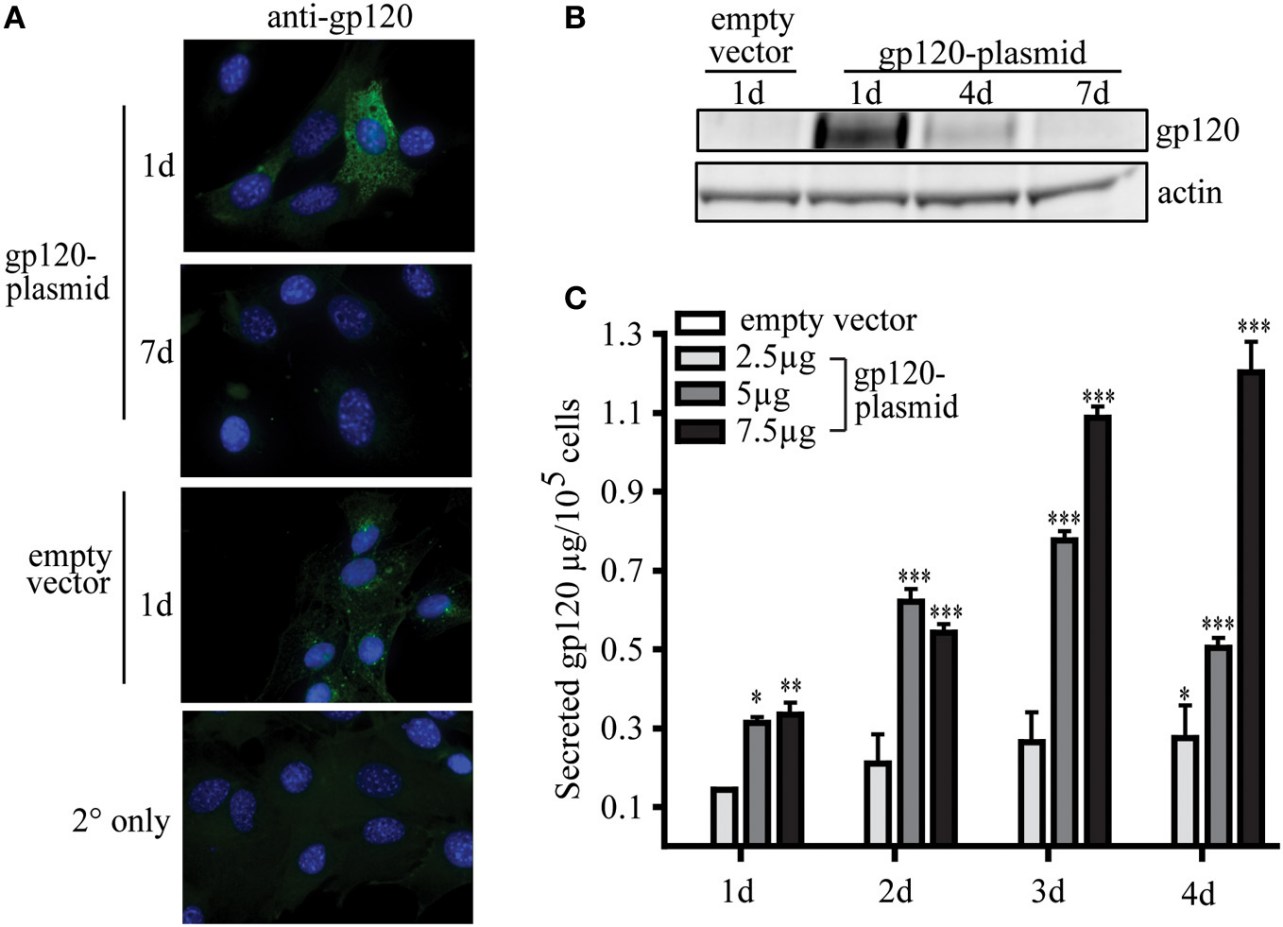
**MSC can be modified to express viral protein gp120.** MSC derived from the bone marrow of C57Bl/6 mice were isolated and validated by the Tulane Center for Stem Cell Research and Regenerative Medicine (New Orleans, LA) as previously described (Ripoll and Bunnell, 2009). A total of 1 × 10^6^ MSC were transfected by electroporation using the Invitrogen Neon system (Carlsbad, CA) with 2.5, 5, or 7.5 μg pSWTK-gp120, or empty vector, pSWTK (generously provided by Dr. V. S. Kalyanaraman of ABL Inc., Kensington, MD) according to the manufacturer's instructions. **(A)** Gp120 immunofluorescence staining of MSC transfected with 5 μg pSWTK or pSWTK-gp120 1 or 7 days post-transfection, and controls using secondary (2°) antibody only, was carried out as previously described at 63X (Tomchuck et al., 2008). **(B)** Western blot analysis of corresponding cell lysates (approximately 25 μg of protein) were probed with anti-gp120 as previously described (Lamarca et al., 2008). **(C)** 1 × 10^5^ transfected MSC were incubated 1–4 days and the harvested cell culture supernatants were analyzed by an HIV-1 gp120 ELISA according to the manufacturer's instructions (ABL Inc.) Data are presented as the mean ± standard error of the mean and analyzed by One Way ANOVA using the Tukey's *post hoc* test (GraphPad Prism Version 4). Statistical significance was determined by comparing pSWTK-gp120 and pSWTK groups. ^*^p < 0.05; ^**^p < 0.01; ^***^p < 0.001.

To date, the ability of MSC to be used as a novel platform for a prophylactic vaccine for infectious disease has not been published. To demonstrate this proof of concept, research conducted by our group has shown that MSC, modified to express a foreign antigen, are sufficient to elicit an antibody-mediated immune response without the need for additional adjuvants or boosting. In our studies, using a plasmid encoding gp120, the glycoprotein from HIV, MSC can be readily modified to secrete a foreign, viral antigen, and stimulate antigen-specific antibody production *in vivo*. These transfected MSC transiently express high levels of gp120 protein intracellularly, with the peak expression 1 day post-transfection (Figures 2A,B). Following transfection, MSC then secrete significant amounts of gp120 protein over 1–4 days in culture (Figure 2C). These expression levels can be controlled in a dose-dependent manner based on the amount of plasmid used during the transfection process. For example, by 2 days post-transfection MSC secreted 2.11 ± 0.73, 6.22 ± 2.98, or 5.41 ± 2.25 μg gp120 per million cells when transfected with 2.5, 5, or 7.5 μg of vector, respectively. Four days post-transfection these cells secreted 2.75 ± 0.81, 5.04 ± 0.252, or 12.03 ± 0.77 μg gp120, respectively. The levels of antigen produced in this transient transfection are sufficient to induce an immunological response from a vaccine standpoint.

**Figure 3:**
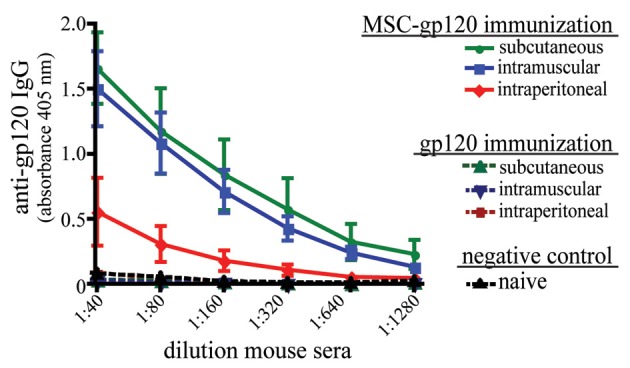
**Modified MSC expressing gp120 promote serum anti-gp120 antibody responses in mice after parenteral immunization.** Groups of five female C57Bl/6 mice 6–8 weeks underwent a single immunization with 1 × 10^6^ MSC transfected with 7.5 μg pSWTK-gp120 (MSC-gp120; solid lines) 16 h post-transfection or 5 μg purified gp120 (a vector-corresponding recombinant protein provided by Dr. V. S. Kalyanaraman; broken lines), with naïve mice serving as a control (black). MSC and gp120 were diluted in DPBS and administered with a 0.5 ml syringe to deliver 100 μl per dose for intraperitoneal and subcutaneous injection, or 50 μl per dose for intramuscular injection. Mice were sacrificed 17 days post-immunization and sera collected. An ELISA for serum anti-gp120 IgG antibodies graphed as 405 nm absorbance versus sera dilution was preformed as previously described (Norton et al., 2011). Animal studies were approved by the Tulane University Institutional Animal Care and Use Committee.

To examine the ability of transfected MSC to elicit an *in vivo* antibody response, C57Bl/6 mice were immunized once by intraperitoneal (IP), subcutaneous (SC) or intramuscular (IM) routes using either MSC-gp120 or purified gp120. Since transplanted MSC persist only for a few days at most *in vivo* (Gao et al., 2001; Abraham et al., 2004; Ohtaki et al., 2008; Prockop, 2009), 5 μg of gp120 was chosen as an amount equivalent to antigen secreted by MSC-gp120, transfected with 7.5 μg of pSWTK-gp120. Similar to other published studies, a single gp120 protein immunization produced no detectable serum antibodies by 17 days post-immunization (Jankovic et al., 1997; McCormick et al., 2001; Lamalle-Bernard et al., 2006) (Figure 3). Mice immunized with MSC expressing an empty vector also did not elicit serum anti-gp120 responses above sera of näive mice (data not shown). However, all mouse groups receiving an MSC-gp120 immunization developed high-titer serum anti-gp120 IgG antibodies regardless of IP, SC, or IM delivery.

## Evidence that MSC can promote adaptive immunity

Our studies with gp120 antigen, in addition to the report by Wei et al., indicate that modified MSC can deliver antigen for protective vaccination against an infectious disease or cancer, in support of our hypothesis. However, the exact mechanisms whereby MSC might be directly influencing the generation of immune responses are unknown. Our experimental data suggest a pertinent role for MSC as more than just a delivery vehicle; gp120 alone (at the estimated dose of MSC-gp120 expression) elicited undetectable humoral responses, similar to previous studies (Jankovic et al., 1997; McCormick et al., 2001; Lamalle-Bernard et al., 2006), while MSC-gp120 induced significant anti-gp120 antibodies after a single immunization.

While MSC are primarily touted for their immunosuppressive properties, several published reports have also directly shown that MSC *promote* adaptive immunity. Table 1 lists publications in which investigators reported MSC-driven activation of T-cells and B-cell responses, mainly through cytokine secretion or antigen-presentation in a variety of experimental settings. In co-cultures, MSC enhanced B-cell proliferation, IL-6 expression, and IgG-secreting plasma cell formation *in vitro*; these B-cell responses could be further augmented with MSC combined with a TLR agonist (lipopolysaccharide or CpG DNA) (Rasmusson et al., 2007; Traggiai et al., 2008). MSC pulsed with tetanus toxoid promoted the proliferation and cytokine expression (IL-4, IL-10, and IFNγ) of a tetanus toxoid-specific CD4 T-cell line (Majumdar et al., 2003; Stagg, 2007; Francois et al., 2009). Similarly, MSC cultured in low ratios (1:100) with lymphocytes in the presence of antigen improved lymphocyte proliferation and CD4 Th17 subset formation, which was partially IL-6 and TGFβ-dependent (Liu et al., 2009). MSC have also been found to express MHC-I and cross-present antigen for expansion of CD8 T-cells both *in vitro* and *in vivo* (Majumdar et al., 2003; Stagg, 2007; Francois et al., 2009).

**Table 1:** **Evidence that MSC can promote adaptive immunity**.

**MSC PROMOTION OF ADAPTIVE IMMUNITY**			
**Experimental setting**	**Defined MSC function**	**Immunologic outcome**	**References**
*In vitro* culture of B-cells/splenocytes and MSC ± TLR agonists (LPS, CpG)	IL-6 secretion	B-cell proliferation IgG secretion	Rasmusson et al., 2007 Traggiai et al., 2008
*In vitro* culture of antigen-specific CD4 T-cells, MSC, and antigen	MHC-II antigen-presentation	T-cell proliferation IL-4, IL-10, IFNγ secretion	Majumdar et al., 2003
	IL-6, TGFβ secretion	Lymphocyte proliferation, Th17 cells	Liu et al., 2009
*In vitro* cultures of splenocytes and MSC; Mouse models of collagen-induced arthritis	IL-6 secretion	Th1 cells lymphocyte proliferation, IL-6, IL-17 secretion	Djouad et al., 2005 Chen et al., 2009
IFNγ stimulation (moderate levels) during *in vitro* cultures of T-cells and MSC; Mouse model of systemic lupus erythematosus	MHC-I/II antigen-presentation, phagocytosis	CD4 and CD8 T-cell proliferation, Anti-tumor CD8+ CTLs	Majumdar et al., 2003; Chan et al., 2006; Stagg, 2007; Francois et al., 2009; Schena et al., 2010
*In vitro* culture with apoptotic cells and CD4 T-cells	MHC-II expression, IL-6 secretion	Th17 cells	Tso et al., 2010

MSC immunoregulation has also been found to be dependent upon external signals. In the presence of inflammatory cytokines or stimulants, MSC therapy, which was previously suppressive, can become immunostimulatory. For example, MSC treated with specific pathogen-associated molecular pattern (PAMP) molecules can become either anti- or pro-inflammatory, depending on the PAMP with which they are treated *in vitro* (Tomchuck et al., 2008; Waterman et al., 2010), reviewed more thoroughly in (Bunnell et al., 2010; Le Blanc and Mougiakakos, 2012). Djouad et al. found that during collagen-induced arthritis, an inflammatory disease setting, transplantation of allogeneic MSC enhanced Th1 immune responses, and IL-6 secretion, which was mimicked *in vitro* by direct TNFα stimulation of MSC (Djouad et al., 2005). A similar study also found MSC administration exacerbated collagen-induced arthritis disease and amplified splenocyte secretion of IL-6 and IL-17 (Chen et al., 2009). Furthermore, pre-treatment of MSC with IFNγ (within a moderate range) upregulates MHC-I and II expression and improves antigen phagocytosis and presentation capabilities, thereby stimulating CD4 and CD8 T-cell proliferation and generation of anti-tumor CD8+ cytotoxic T-lymphocytes (CTLs) (Majumdar et al., 2003; Chan et al., 2006; Stagg, 2007; Francois et al., 2009; Schena et al., 2010). In another study, co-culture of MSC with apoptotic cells, which mimics conditions of rheumatoid arthritis, induced Th17 cells through IL-6 expression on MHC-II expressing MSC (Tso et al., 2010).

These studies support our hypothesis that MSC can be used to as a novel vaccination platform generating protective immunity. They also suggest mechanisms that may be involved during modified MSCs vaccination besides antigen delivery, including cytokine secretion and antigen presentation (Figure 1B). While not always explicitly required, the enhanced promotion of immunity by MSC seen with cytokine or PAMP treatment indicates that vaccine antigens that are highly immunogenic may direct a more immunostimulatory phenotype of the MSC used for vaccination. For example, MSC modified to express a bacterial or viral TLR ligand, in conjunction with other pertinent microbial antigens, may be able to promote even higher levels of protective antigen-specific immunity than microbial antigens by themselves. It is also tempting to speculate that the modification process combined with the expression of any immunogenic antigen may provide some sort of “inflammatory” signal to the MSC that could positively impact subsequent generation of vaccination responses.

## Knowledge gaps and future directions

The idea of MSC as a novel vaccine approach is clearly still in its infancy and many knowledge gaps exist before this strategy could ever be practically realized. In particular, while the potential for MSC as a delivery system is tremendous, in depth evaluation is needed into how many foreign or cancer antigens can be effectively expressed by MSC. It will also be important to perform a more detailed analysis on the impact of MSC vaccination responses on antigen-specific immunity, including humoral and cellular responses, induction of long-term memory, etc. As described above, it will be critical to establish the mechanism(s) whereby modified MSC promote vaccine immunity. While clearly MSC can effectively express and secrete vaccine antigens, these MSC might be more directly enhancing immune responses through antigen presentation and/or inflammatory cytokine expression, as seen in previous non-vaccination studies (Table 1). It is particularly exciting that MSC may be able to enhance immune responses by direct antigen presentation to T-cells. However, to date there has been no examination of whether this happens during a vaccination setting and future scientific examination of MSC for antigen presentation of vaccine antigens is warranted. This possible ability of MSC to act as conditional APCs, but with less risk for GvHD, makes them an attractive alternative to other cellular-based vaccinations.

One aspect of transfected MSC for vaccination is the difference in immunologic responses between stable-transfection strategies versus transient-transfection. Logic would indicate that a transient transfection might offer the best safety profile, without the worry of cell persistence, tumorigenicity etc. In addition, since MSC can have alternative immunomodulatory functions during inflammatory conditions and home to inflamed sites (at least when given intravenously), it will also be important to ascertain how effective an MSC vaccination will be when the host recipient is under any inflammatory disease setting. Therefore, a careful examination of the safety of MSC vaccination will also need to be evaluated.

## Concluding remarks

In summary, MSC have unique abilities that could enable their use as a novel vaccine delivery method. These include (1) protection from allogeneic host responses (GvHD), (2) ease of production attributes including ability to be expanded and modified *ex vivo* for transient or stable transfection before *in vivo* administration, (3) ability to act as delivery vehicle/depot for antigen release over several days, and (4) initiation and possibly direct stimulation of antigen specific immune responses to these antigens *in vivo*.

Such a MSC platform is theoretically capable of expressing hundreds of proteins, thereby generating a broad array of epitopes with correct post-translational processing, mimicking natural infection. By stimulating immunity to a combination of epitopes, it may be possible to develop prophylactic and even therapeutic vaccines to major global health diseases, like HIV, where traditional vaccination approaches have failed. While modified MSC delivery is unconventional, their unique properties may indeed be able to serve as an innovative vaccine platform.

### Conflict of interest statement

The authors declare that the research was conducted in the absence of any commercial or financial relationships that could be construed as a potential conflict of interest.
